# Unveiling Rare Complications: Pancreatic Pseudocysts and Monomicrobial Non-neutrocytic Bacterascites in Decompensated Cirrhosis

**DOI:** 10.7759/cureus.55909

**Published:** 2024-03-10

**Authors:** Harshitha Reddy, Rushikesh H Dhondge, Sunil Kumar, Sourya Acharya

**Affiliations:** 1 Internal Medicine, Jawaharlal Nehru Medical College, Datta Meghe Institute of Higher Education and Research, Wardha, IND

**Keywords:** case report, cirrhosis., dysphagia, pseudocyst, pancreatitis

## Abstract

One frequent side effect of chronic pancreatitis is pancreatic pseudocyst. Abdominal pain and vomiting are common complaints that the patient presents with. However, atypical manifestations of pancreatic pseudocyst still confound medical professionals worldwide, making identification challenging and ultimately increasing the risk of fatal consequences. We describe the case of a 41-year-old man who had decompensated liver cirrhosis linked to alcohol intake. The patient presented with dysphagia and yellowish skin discolouration, which, upon further investigation, turned out to be peripancreatic pseudocysts extending into the mediastinum. Diagnostic challenges arose due to the rare occurrence of a pancreatic pseudocyst located in the mediastinum. Patient was treated with a culture-sensitive antibiotic and on follow up he was doing well.

## Introduction

Pancreatic pseudocysts are known complications of pancreatitis, typically found within or adjacent to the pancreas. However, extra-pancreatic locations, especially in the mediastinum, are exceedingly rare. Cirrhosis of the liver can lead to various complications, but its association with pancreatic pseudocysts in atypical locations is rare [1].

Pancreatic secretions rich with amylase escape along the paths of least resistance when inflammatory damage disrupts the pancreatic ducts. Pancreatic ascites are caused by anterior disruptions, but thoracopancreatic fistulae might result from posterior disruptions [2]. Due to its unusual placement, mediastinal pseudocysts can manifest a wide range of symptoms, including difficulty in swallowing, palpitations and chest pain. In severe cases, there is pericardial effusion, cardiac tamponade, and respiratory failure [3,4]. When people with chronic liver disease have a negative fluid analysis for spontaneous bacterial peritonitis but a positive culture, they have a condition called monomicrobial non-neutrocytic bacterascites (MNB) [5].

## Case presentation

Arriving at the hospital, a 41-year-old male complained of difficulty in swallowing for 15 days, initially for solid foods and progressed to dysphagia for a liquid diet. He also had complaints of breathlessness, which gradually progressed from New York Health Association Grade II to IV over 10 days. The patient also complained of yellowish discolouration of the eye and abdominal distension for one month. The patient has been a chronic alcoholic for the past 35 years, with the intake being approximately 700 ml/day, and his last intake was three months back. He denied any history of diabetes mellitus and systemic hypertension.

On general examination, his pulse rate was 136bpm, blood pressure was 90/60mmHg, oxygen saturation level when breathing ambient air was 84%, and respiratory rate was 36 cycles/min. The patient had pallor, icterus and bilateral pedal oedema. Respiratory system examination indicated dull percussion notes and absent air entry in the inframammary, lower axillary, and infra scapular areas in both lung fields. Abdominal examination revealed raised local temperature, guarding in the upper abdomen, with a positive fluid thrill and shifting dullness. Other system examinations were within normal limits. His routine laboratory investigation is highlighted in Table 1.

**Table 1:** Laboratory parameters of the patient

Laboratory investigations	Value in the patient	Biological Reference Range
Haemoglobin	5.6	13-15 g/dl
Total Leucocyte Count	3300	4000-11000/ cumm
Platelet	90,000	1,50,000-4,50,000/cumm
Mean corpuscular volume	102.3	79-100 fL
Urea	46	9-20 mg/dl
Creatinine	1.3	0.6-1.2 mg/dl
Sodium	135	135-145 mmol/l
Potassium	3.2	3.5-5.1 mmol/l
Alkaline Phosphatase	122	38-126 unit/l
Alanine Transaminase	245	<50 U/l
Aspartate Transaminase	302	17-59 U/L
Total Protein	4.2	6.2- 8.3 gm/dl
Albumin	2.2	3.4-5 gm/dl
Total Bilirubin	0.7	0.2-1.2 mg/dl
Globulin	2.0	2.3-3.5 gm/dl
Erythrocyte Sedimentation Rate	80	<15 mm/hr
Activated Partial Thromboplastin Clotting Time	54.1	29.5 Control
Prothrombin Time	15.6	11.9 Control
International Normalized Ratio	2.09	0.8-1.2
Amylase	1200	40 -140 u/l
Lipase	3000	0-70u/l

**Figure 1:**
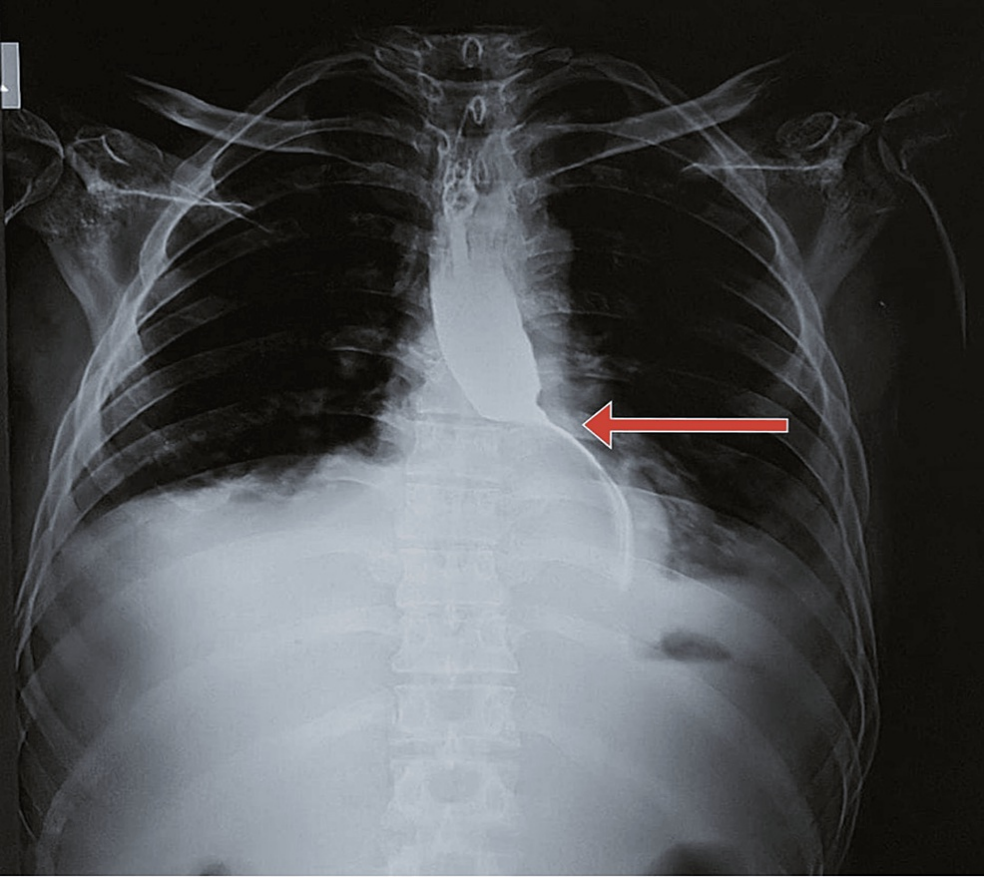
Narrowing at the lower esophageal sphincter junction due to compression of mediastinal pseudocyst (red arrow)

Pleural fluid analysis revealed transudate fluid with an amylase level of 1200 U/L and a lipase level of 2100 U/L. His peritoneal fluid analysis indicated a total leucocyte count of 25 cells/cumm, and culture revealed Pseudomonas aeruginosa sensitive to ceftazidime. His viral markers for hepatitis B virus, hepatitis C virus, and human immunodeficiency virus were non-reactive. A barium swallow was done as the patient was complaining of dysphagia, and it showed narrowing at the lower oesophagal sphincter junction (Figure 1). 

**Figure 2:**
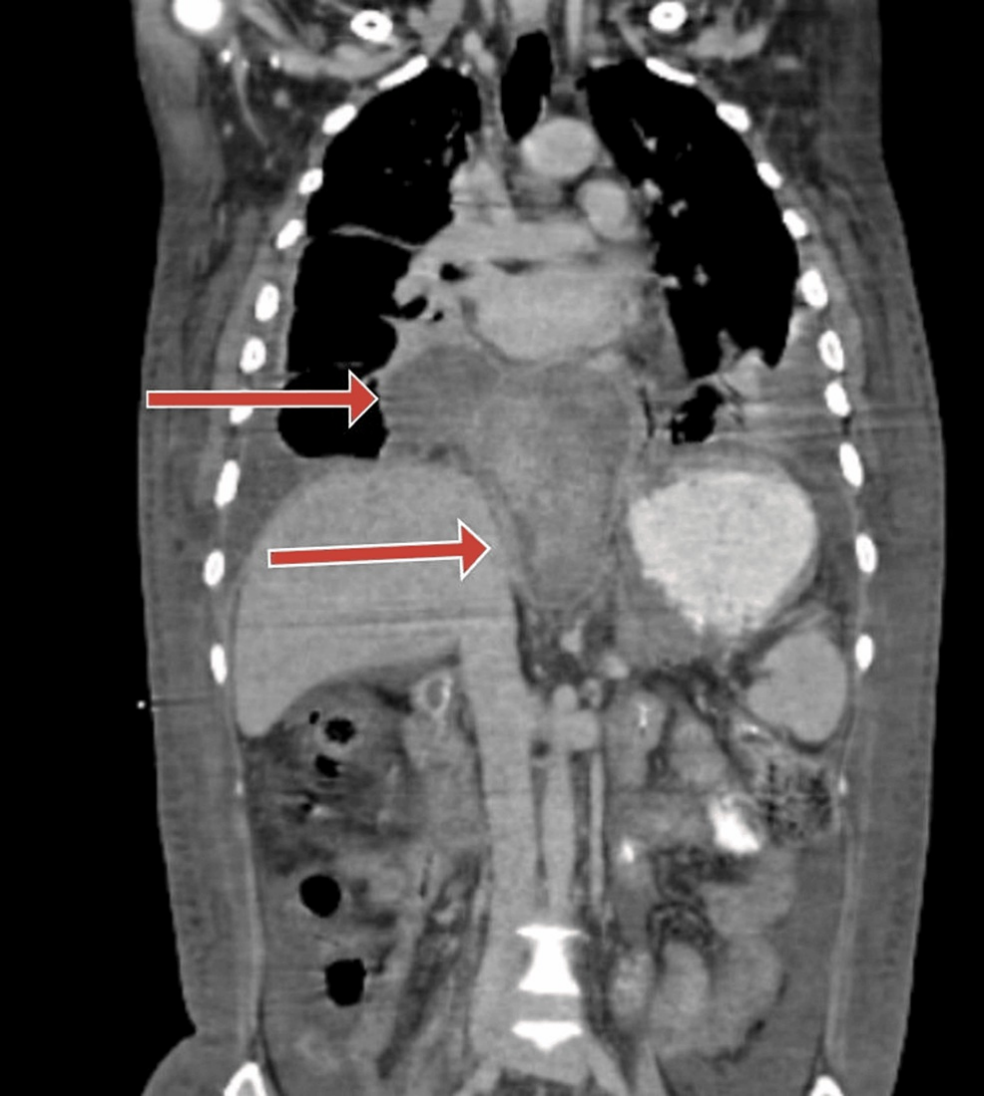
Contrast-enhanced computed tomography imaging of abdomen revealed the pancreatic pseudocyst with mediastinal extension (red arrows).

Contrast-enhanced computed tomography (CT) imaging of the abdomen revealed the pancreatic pseudocysts having mediastinal extension (Figure 2). 

**Figure 3:**
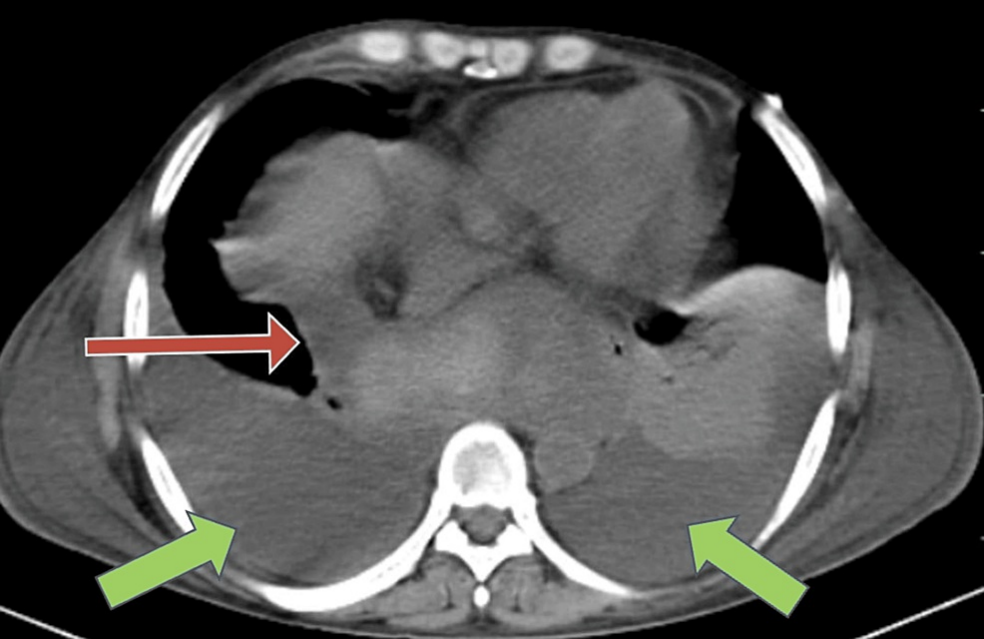
A pancreaticopleural fistula on the right side (red arrow) and bilateral pleural effusion (green arrows) were seen on the computed tomography scan of thorax

A pancreaticopleural fistula on the right side (red arrow) and bilateral pleural effusion (green arrows) were seen on the CT scan of thorax (Figure 3).

The patient was started on injectable ceftazidime, based on the culture report, which revealed growth of Pseudomonas sensitive to ceftazidime. The patient was also given injectable thiamine and tablet rifaximin. Gatroenterologist opinion was taken for endoscopic drainage of pseudocyst, but patient and his relative denied due to financial constraints. Patient was asked to follow up and on one-month follow-up patient was doing well.

## Discussion

Pseudocysts and pancreatic necrosis are examples of known complications that follow either acute or chronic pancreatitis. Mediastinal pseudocyst is an uncommon consequence. Because there are more pulmonary signs and symptoms than indicators of pancreatic illness, the presentation is frequently unclear. Dyspnea and chest or abdominal pain are the most frequently occurring presenting symptoms. Thoraco-pancreatic fistulas are formed when there is reduced resistance to the mediastinum due to the presence of inflammation and fibrosis along the conventional peri-pancreatic gaps. Based on the location of the fistula's termination, thoracopancreatic fistulas are classified as pancreaticopleural, mediastinal pseudocyst, pancreaticobronchial, and pancreaticopericardial [6]. Most fluid enters the posterior mediastinum through the oesophageal and aortic hiatuses. However, this fluid will spread into the anterior and middle mediastinum via the foramen of Morgagni or the inferior vena cava hiatus [7]. Pleural fluid analysis and several radiologic procedures are usually used to establish the diagnosis. Usually, the pleural fluid has a high concentration of lipase and amylase. Patients with pancreaticopleural fistulas may have mean amylase levels in their pleural fluid that are more than 10,000 IU/L [8]. 

Although ultrasound is a readily available diagnostic tool for peripancreatic pseudocysts, its usefulness in identifying mediastinal pseudocysts is limited due to its location. When pancreatic abnormalities are suspected, CT should be the first abdominal imaging scan performed. It is good at identifying these conditions. Additionally, CT can provide insight into the relationship between the pancreatic and the mediastinal cystic formations. Furthermore, magnetic resonance cholangiopancreatography (MRCP) provides the greatest definition of ductal morphology, including disruption, dilatation, stricture, and communication with pseudocyst. When evaluating a mediastinal mass, endoscopic ultrasonography (EUS) is a crucial diagnostic technique that can aid in therapy planning and enable guided cyst aspiration and drainage. With endoscopic retrograde cholangiopancreatography (ERCP) and MRCP, the fistula can be identified [9]. 

The size of the pseudocyst, the ductal architecture, the underlying aetiology, the clinical symptomatology, and the availability of specialists all affect how mediastinal pseudocysts are managed. It is uncommon for a mediastinal pseudocyst to spontaneously resolve under conservative care [10]. For asymptomatic individuals with tiny cysts, conservative treatment with somatostatin analogues and complete parenteral nutrition can be effective. Drainage procedures can be performed percutaneously with CT or ultrasound guidance or endoscopically utilizing the transesophaegal, transgastric, or transpapillary approach. When compared to percutaneous drainage, endoscopic therapy has the benefit of a lower risk of complications.Patients with big cysts or those who are symptomatic should have drainage treatment. Surgery should be used to treat pseudocysts that are associated with infection, obstruction, rupture, or bleeding. Surgery such as distal pancreatectomy, pancreatic head resection, cysto jejunostomy, cystogastrostomy, or Puestow technique is done [11].

Non-neutrocytic bacterascites patients may lack signs of peritoneal irritation, and ascitic fluid analysis may reveal a low neutrophil count (<250 cells/mm^3) despite positive bacterial cultures. Management of non-neutrocytic bacterascites involves empirical antibiotic therapy directed against common pathogens in cirrhotic patients, such as Gram-negative bacteria and staphylococci. The prognosis of non-neutrocytic bacterascites largely depends on the underlying severity of liver disease, the virulence of the infecting organism, and the promptness of antibiotic therapy. Delayed diagnosis and inadequate treatment may result in systemic complications, such as sepsis or hepatic decompensation [12].

## Conclusions

This case highlights the diagnostic intricacies encountered when dealing with rare presentations of pancreatic pseudocysts, especially in patients with underlying cirrhosis and ascites. A multidisciplinary approach involves imaging and invasive procedures like EUS and ERCP. The coexistence of pancreatic pseudocysts in the mediastinum and cirrhosis of the liver, in this case, posed diagnostic and management challenges. The association between chronic alcoholism, pancreatitis, and subsequent pseudocyst formation and its extension into the mediastinum could explain the patient's clinical presentation. Additionally, the compromised liver function influenced the management strategy, necessitating a cautious approach during invasive procedures due to the increased risk of bleeding.

## References

[1] Dronamraju S, Gupte Y, Pawar T, Acharya S, Kumar S (2021). Acute pancreatitis with large infected pseudo pancreatic cyst in a post-partum female - case report. J Evolution Med Dent Sci.

[2] Moorthy N, Raveesha A, Prabhakar K (2007). Pancreaticopleural fistula and mediastinal pseudocyst: an unusual presentation of acute pancreatitis. Ann Thorac Med.

[3] Raut SS, Acharya S, Kumar S, Deolikar V, Kothari M (2024). Recurrent acute-on-chronic pancreatitis in a chronic alcoholic with pancreatic divisum: a complex case. Cureus.

[4] Shah P, Bagga C, Talwar D, Kumar S, Acharya S (2022). Mediastinal eventration of a pseudocyst of pancreas presenting as acute shock syndrome: expecting the unexpected. Cureus.

[5] Neto MB, Chedid V, Berbari E (2017). Monomicrobial non-neutrocytic bacterascites due to clostridium: to treat or not to treat?. Am J Gastroenterol.

[6] Segamalai D, Abdul Jameel AR, Kannan N, Anbalagan A, Duraisamy B, Raju P, Devy Gounder K (2017). Mediastinal pseudocyst: varied presentations and management—experience from a tertiary referral care centre in India. HPB Surg.

[7] Altasan T, Aljehani Y, Almalki A, Algamdi S, Talag A, Alkattan K (2014). Pancreaticopleural fistula: an overlooked entity. Asian Cardiovasc Thorac Ann.

[8] Sinukumar S, Naik S, Khurjekar D, Munde Y, Bhosale S (2020). Pancreaticopleural fistula after cytoreductive surgery and HIPEC for Pseudomyxoma peritonei—a rare presentation and rare complication. Indian J Surg Oncol.

[9] Dąbkowski K, Białek A, Kukla M (2017). Mediastinal pancreatic pseudocysts. Clin Endosc.

[10] Talwar D, Kumar S, Acharya S, Madaan S, Hulkoti V (2021). Intractable hiccups in a young male: is it a tell-tale sign of pseudocyst of pancreas?. Cureus.

[11] Aghdassi AA, Mayerle J, Kraft M, Sielenkämper AW, Heidecke CD, Lerch MM (2006). Pancreatic pseudocysts--when and how to treat?. HPB (Oxford).

[12] Haider S, Gupta R, Sood A, Kanitkar A, Saydain G (2016). Monomicrobial non-neutrocytic bacteriascites due to salmonella enteritidis: a case report and literature review. J Med Cases.

